# Unrestricted Learning Opportunities for Trainees in Behavior Analysis: A Survey of Current Practices

**DOI:** 10.1007/s40617-024-00931-3

**Published:** 2024-04-11

**Authors:** Clare J. Liddon, Madison Crandall, Regan Weston

**Affiliations:** Department of Counselor Education and Human Services, University of Dayton, Dayton, OH USA

**Keywords:** Unrestricted activities, Fieldwork, Barriers, Trainees, Supervisors

## Abstract

Individuals seeking certification as a board certified behavior analyst (BCBA) by the Behavior Analyst Certification Board (BACB) must meet certain eligibility requirements. In addition to passing the BCBA examination, such requirements include completion of a master’s degree, behavior-analytic coursework, and supervised practical fieldwork. In accruing fieldwork hours, trainees must be provided with the opportunity to complete unrestricted activities. The BACB defines unrestricted activities as “. . . those that are most likely to be performed by a BCBA,” and requires that 60% of fieldwork hours are comprised of these activities (BACB, 2022b). Fieldwork hours may be accrued across a number of different host sites (e.g., hospital units, schools, community locations), with each host site having different day-to-day responsibilities affecting how these opportunities are provided. Therefore, exploration of the provision of these opportunities and the barriers to providing these opportunities is warranted. The current study sought to determine the current practices involved in provision of opportunities to gain fieldwork experience hours towards BCBA certification; in particular, practices related to unrestricted fieldwork activities. Results indicate that, although unrestricted learning opportunities are often provided to trainees, contingencies present within the day-to-day operations of a clinical environment can be hampering. A discussion of the implications of these barriers and potential solutions are included.

One of the requirements for individuals aspiring to become board certified behavior analysts (BCBA) is to obtain practical, hands-on experience. The Behavior Analyst Certification Board (BACB) provides parameters for the types of activities an individual can count towards this fieldwork experience (BACB, 2022b). Those engaged in accruing such field work experiences are referred to as trainees. Two distinct experience types are defined in the *BCBA Handbook*: (1) restricted activities, and (2) unrestricted activities (BACB). Restricted activities include experiences in which a trainee is delivering therapeutic or instructional procedures directly with a client (BACB). These activities can only account for up to 40% of a trainee’s total fieldwork experience (i.e., up to 800 hours in Supervised Fieldwork). The remaining 1,200 hours should be comprised of unrestricted activities. The BACB defines unrestricted activities as “those that are most likely to be performed by a BCBA” (p. 16). The ratio of restricted to unrestricted fieldwork hours was modified (from a 50% minimum requirement of unrestricted hours) based on the recommendations of a subject matter expert committee. The decision for these changes was based on rapid growth of the field in addition to consumer appeals for more rigorous supervised experience requirements, subject matter experts’ direct experiences with recent graduates in the field, and increases in the rigor of other BACB standards (BACB, 2017a). This change allows for aspiring behavior analysts to gain more experience in unrestricted tasks during their training.

The importance of unrestricted hours is emphasized within the current requirements in several ways. The percentage of unrestricted hours is not limited to 60% of the trainee’s total fieldwork hours. This is rather a minimum number of unrestricted hours to be accrued. In addition, in specifying a maximum allowed number of restricted hours, the BACB does not actually require trainees to accrue restricted hours. In other words, it would be acceptable for a trainee’s entire fieldwork experience to be comprised of unrestricted activities. By placing a limit on the total duration of restricted activities, the BACB attempts to ensure that a trainee is provided with sufficient opportunities to develop the skills needed to function as a BCBA. Once a behavior analyst is certified, the majority of their time will be spent performing unrestricted tasks, thus trainees should become proficient before certification.

The minimum requirements provided by the BACB help to ensure that trainees are provided with some experiences that support the development of the skills needed to become competent practitioners. Beyond these minimum requirements, there are several aspects of the supervised fieldwork experience to be considered, including the quality of the supervised fieldwork experience.

## Quality of Fieldwork Experiences

Expectations for the quality of the supervisory experience are outlined in the *Ethics Code for Behavior Analysts* (ECBA; BACB, 2022c). Section 4 in the *Code* describes a BCBA’s responsibility to their supervisees and trainees. These guidelines relate to both clinical supervisees (i.e., those not accruing fieldwork hours) and trainees. A second resource highlighting the importance of high-quality supervision is the BACB 5th edition *Task List* (BACB, 2017b), which includes a list of the knowledge content areas and skills found on the BCBA exam. An entire subsection of the task list includes several items directly related to supervising others which trainees should be familiar with to prepare for the board certification exam (BACB, 2017b). Further, these requirements will endure, with the section “I. Personnel Supervision and Management” of the BACB’s 6^th^ edition *Test Content Outline* (TCO; BACB, 2022a), set to replace the 5^th^ edition *Task List* in 2025. Although these resources guide higher-level considerations related to the supervisory experience (e.g., meeting general requirements, monitoring performance, maintaining appropriate documentation) as well as the skills needed to effectively carry out these responsibilities, specific details of what the supervisory experience should look like are not provided.

The burden of determining what types of activities count towards a trainee’s fieldwork experience falls to the person overseeing the fieldwork experience (i.e., the supervisor). The BACB cites unique goals, situational variables, and varied populations and settings as to why they do not provide specific guidance beyond the general, minimum requirements (BACB, 2022b). This means that it is the supervisor’s responsibility to develop performance goals, design and implement appropriate fieldwork opportunities, evaluate the trainee’s progress towards meeting their goals, and provide feedback on the trainee’s performance. This flexibility in determining what a trainee’s experience will look like may lead to a wide range of experiences across supervisors. As a result, researchers and practitioners have sought to identify effective strategies to deliver quality supervised experiences.

## Evidence-Based Supervision

In recent years, there has been an increased focus on the quality of supervision being provided to trainees within the field. Expectations delineated by the BACB as well as an increase in research focused on examining practitioners’ knowledge, experience, and comfortability with providing supervision have led to the development and distribution of a variety of resources for those providing supervision.

In 2016, *Behavior Analysis in Practice* (*BAP*) included a special issue that was focused on supervision. This issue provided some of the first peer-reviewed resources for supervisors in the field. Articles focused on ethical considerations related to supervision (e.g., Turner et al., 2016), systematic approaches for offering supervision in group settings (e.g., Valentino et al., 2016), and general guidelines and best practices for arranging supervisory experiences (e.g., Hartley et al., 2016) were included.

Since the publication of the *BAP* special issue, various textbooks have been published that provide ways to structure supervision in terms of topics to discuss, competencies to focus on, and activities for the trainee to complete. Kazemi et al. (2018) offer a handbook that allows supervisors and trainees to work through a formal supervised experience by completing a series of competencies directly related to items from the 5th Edition *Task List* (BACB, 2017b). In addition, the handbook guides trainees on how to identify a suitable fieldwork site and enter into a quality supervisory relationship with potential supervisors. LeBlanc et al. (2020) took a slightly different approach in their supervision textbook, which examines a wide array of topics ranging from how to establish quality professional relationships to developing skills related to cultural competence. The accompanying activities throughout the book provide the supervisor and trainee with opportunities to engage in higher-level discussions related to each of the topics, define goals, and evaluate progress towards those goals over time.

Researchers have also provided guidance on how to structure supervision in various ways. Garza et al. (2018) provide a systematic approach to supervising those aspiring to become BCBAs by offering a set of tools that supervisors can use to ensure they are engaging in empirically based supervisory practices. The authors described a five-phase supervision process that consists of establishing a supervisory relationship, conducting skills assessments, providing training, ongoing performance monitoring, and ending the supervisory relationship (Garza et al., 2018). Another example came in response to the COVID-19 pandemic and the need to evaluate the effects of virtual supervision (Simmons et al., 2021). Simmons et al. looked at the acceptability and feasibility of providing supervision through virtual means by examining aspects such as the supervisor’s availability, responsiveness, and preparedness in addition to outcomes such as effectiveness, comfort, and trainees’ contributions and preparedness. Although a preference for in-person or hybrid supervision models was identified, general satisfaction as well as the perceived effectiveness of virtual supervision was also indicated by the results. These findings suggest there may be flexibility in the mode of supervision without compromising the quality of the supervision experience for trainees.

Published resources are increasing in number as the focus on quality and evidence-based supervision continues to expand. These resources provide supervisors and trainees with an array of tools that can guide the development of quality supervision experiences.

## Other Factors Affecting the Supervisory Experience

Once evidence-based experiences have been identified and defined, some supervisors must also consider whether their trainees have the necessary credentials to perform these activities. This consideration is more relevant in clinical sites that accept payments from third-party payers (e.g., insurance companies, government agencies).

The 2019 Current Procedural Terminology (CPT) codes for Adaptive Behavior Services dictate how billable time (i.e., time reported to, approved, and funded by third-party payers) should be spent. One consideration that may affect trainees’ ability to participate in unrestricted opportunities is that only face-to-face time is reported for billing purposes (American Medical Association [AMA], 2018). Tasks related to a service that must be completed before or following the interaction with a patient or client (e.g., reviewing client records or writing a progress note) must be bundled with the direct service provided. In other words, the work completed outside of the face-to-face time is factored into the reimbursement rate. Although there are limited situations in which a qualified health professional can bill for the direct monitoring of services provided by a technician during face-to-face time with a client, they cannot bill for providing supervision when the patient is not present. This creates the need for careful consideration of how supervisors allocate their time with trainees to ensure appropriate experiences are provided while adhering to the requirements of any relevant third-party payer policies.

Another noteworthy distinction that potentially affects trainees’ unrestricted opportunities is how the terms *direction* and *supervision* are defined with regard to CPT codes. *Direction* relates to the supervisor’s (or qualified health professional’s) direct oversight of observing a technician implementing a patient’s protocol. This typically falls under the restricted activity type according to the *BCBA Handbook*. On the other hand, *supervision* relates more to those skills that would fall under unrestricted activities such as practicing in a competent, professional manner, and continuing to develop their knowledge and skills. Activities that relate directly to meeting requirements for obtaining professional credentials are generally not categorized as billable services using CPT codes. These considerations add to the complexity of supervisors providing unrestricted opportunities to trainees. It is important to identify ways to offer such opportunities that align with professional ethical guidelines, state and national employment regulations, as well as any third-party payer policies.

The task of offering high-quality supervision for trainees working towards BCBA exam eligibility comes with many considerations. Supervisors must be familiar with the eligibility requirements and are proficient in developing fieldwork experiences that meet the BACB’s requirements. Sellers et al. (2019) shed light on practitioners’ current supervisory practices. Through the use of descriptive statistics, the authors noted several areas supervisors provide consistent and quality experiences such as using contracts to set clear expectations and utilizing a range of performance evaluation strategies. In addition to collecting information on supervisory practices, Sellers et al. also sought to identify barriers to implementing those practices. Some of the barriers noted included a lack of access to examples and a lack of knowledge on how to measure or teach trainees to respond to feedback, time management, organization, and interpersonal communication. One of the most frequent barriers identified in the survey was a lack of time to prepare for meetings and to create systems to track mastery of knowledge and skills (Sellers et al., 2019). These barriers relate directly to some of the items in the ECBA (i.e., items 4.08 “Performance Monitoring and Feedback” and 4.10 “Evaluating Effects of Supervision and Training”; BACB, 2022c*)* as well as items in the 5th Edition *Task List* (i.e., I-5 “Use performance monitoring, feedback, and reinforcement systems,” I-7 “Use function-based strategies to improve personnel performance,” and I-8 “Evaluate the effects of supervision”; BACB, 2017). These findings indicate a need for increased emphasis on the development and progress monitoring of unrestricted skills among individuals aspiring to become BCBAs.

## Supervision in Other Fields

Behavior analysis is not the only discipline that must provide training using a scientist-practitioner model in the context of service delivery. With the apparent need to increase the progress monitoring of those aspiring to become BCBAs, we may find it useful to look to other, more established disciplines that provide human services for solutions to similar challenges. Supervised experience is a common way for students and early-career practitioners to gain real-world experience in many disciplines. The fields of psychology and medical education both include several types of supervised opportunities before supervisees are able to practice independently. Medical students, for example, complete 1–2 years of didactic coursework, 2 years of unpaid supervised experience (i.e., clerkship), and two segments of the U.S. Medical Licensing Examination (USMLE) in medical school (Federation of State Medical Boards [FSMB], 2022). Once the degree is awarded, physicians are required to pass the final portion of the USMLE before they are able to obtain a state training license or unrestricted state medical license (this depends on the location of their residency program). This license allows the resident physician to practice as a licensed physician under the supervision of a faculty member at their training site.

Although licensure requirements vary by state, several professional boards and organizations (e.g., Association of American Medical Colleges, FSMB, Liaison Committee on Medical Education,) have developed general guidelines and requirements to create a streamlined process for medical students and resident physicians across state boundaries (Accreditation Council for Graduate Medical Education [ACGME], 2022). After the completion of a residency (as well as a fellowship for some specialties), physicians are able to apply for their respective board exams. Board certification, although not necessarily required, allows physicians to demonstrate competency within a specialized area. ACGME requires that medical doctors complete a minimum of 3 years supervised experience, with some specialties requiring up to 7 years of supervised residency training following graduation from medical school (ACGME, 2022).

Likewise, students pursuing their doctoral degree to become licensed psychologists are required to complete several supervised experiences. As the compulsory credential for psychologists is a license, each state stipulates its own requirements (Association of State & Provincial Psychology Boards [ASPPB], 2022). The requirements by state vary greatly in terms of how many hours are required, what type of experience is accepted as supervised experience, and which examinations are required (e.g., EPPP part 1 and 2, oral examination, jurisprudence exam). The ASPPB provides guidelines and expectations of what the supervised experiences should entail (ASPPB, 2020). The three types of supervised experiences psychology students complete include practicum hours, internship hours, and postdoctoral hours.

Practicum training is completed within a graduate program, providing the supervisee with real-world experience in a closely monitored and heavily supervised setting. The doctoral internship is completed following the completion of all coursework and practicum hours and is at least 1 year in duration. The internship allows the trainee to learn intermediate to advance skills and allows for a shift in the amount of monitoring and supervision as the supervisee demonstrates competence. The postdoctoral fellowship is completed following the completion of the internship and after a doctoral degree is awarded. This fellowship is the final level of formal education for psychologists. This experience focuses on the trainee’s professional identity and advanced applied competencies rather than on developing competence in basic skills. Monitoring and supervision are again scaffolded as the trainee advances through the fellowship. Each state determines whether all three supervised experiences are required or not and how those hours can contribute to the number of hours required for licensure (ASPPB, 2022).

The brief summaries of medical and psychology education provided here illustrate that other fields using a medical or science-practitioner model of training maintain standards similar to, if not more rigorous, than those delineated by the BACB, and do so in the setting of service delivery. These summaries also provide important context for understanding how current supervisors in behavior analysis are providing opportunities for practice in training, as well as barriers to being able to provide such opportunities will benefit current and future supervisors alike.

## Purpose

As noted previously, unrestricted activities are the primary skills required of a competent behavior analyst and as such, should be the primary focus of training. In addition to ensuring that quality experiences for trainees to develop unrestricted skills are provided, fieldwork supervisors are also tasked with the responsibility of supporting clients’ individual needs while adhering to third-party payer contingencies that may exist within their agencies. Each of these variables is associated with different goals and outcomes; thus, it is important to examine how they interact and affect one another. It is important to note that supervision may oftentimes be of secondary importance to the supervisor (Garza et al., 2018). This means that providing quality experience may take lower priority than ensuring clients’ needs are met, particularly when third-party payer contingencies rely on direct interactions with the client. Regardless of this potential discrepancy of priorities, providing effective and meaningful experiences for trainees is of paramount importance for the development of the field. As Sellers et al. (2019) noted, if future supervisors are not trained to effectively supervise, the risk for trainees to adopt the same faulty repertoires is increased. As such, identifying ways to provide quality unrestricted opportunities should be a primary focus. Likewise, illuminating potential barriers to providing such opportunities is equally important to be able to address issues that exist within supervisory experiences. The purposes of the current study are to collect information on how qualified supervisors are currently providing unrestricted learning opportunities for their trainees and to identify barriers they encounter when offering these experiences.

## Method

### Survey Development and Distribution

A survey was developed and piloted by individuals with knowledge of BACB fieldwork requirements, including practices that make up a quality, unrestricted learning experience and common factors that might affect completion of such practices. The individuals that designed and piloted the survey were excluded from participation. The survey was designed within Qualtrics (2022), computer software with the capability to collect anonymous responses via the Internet.

The survey had three primary sections: inclusion or exclusion criteria, demographic information, and information on current practices in offering unrestricted activities to trainees. All questions were selection-based, with the exception of the final question. The final question was presented in an open-ended format, allowing respondents to freely enter text. Selection-based questions were offered in multiple formats: multiple choice with single answer selection, and multiple choice with multiple answer selection. Some multiple-choice options allowed participants to select “Other,” and instructed the participant to specify the answer in an open-ended box. Though multiple answer selections prevent an evaluation of mutually exclusive responses, they were chosen for use in the survey to adequately approximate the supervision environment. That is, it is likely multiple factors might affect a supervisor’s ability to offer unrestricted learning opportunities, and the multiple choice with multiple answer selection questions were designed to reflect this. Within the survey, trainees were referred to as “supervisees.”

Survey distribution took place via email and social media. Emails were distributed through a public listserv, a mass email service, and personal communication. An invitation letter with an overview of the purpose of the study and a link to the survey was attached within the emails or social media postings. Participant recruitment and corresponding data collection took place from July 14 through September 2, 2022; that is, the survey opened and closed on these dates.

### Quantitative Data Analysis

Quantitative data were analyzed using the *Statistical Package for the Social Sciences* (SPSS) Version 28. For all analyses, data were only included for respondents who completed the entire survey and reported they provided supervision (*n* = 182). The primary focus of data analysis was to provide descriptive information of the current unrestricted activity opportunities offered and barriers for completing them at various types of fieldwork sites. Because the survey was meant to collect extensive information about supervisors’ practices, reporting results for each variable was not practical. Complete data are available from the authors.

A secondary purpose was to identify potentially relevant variables that significantly affect either the unrestricted activities available or the barriers to complete them. We had no a priori predictions for what variables might be significant, thus these analyses should be interpreted as hypothesis-producing. Future studies would need to replicate these analyses with a priori research questions, directional hypotheses, and samples explicitly recruited to represent all relevant subgroups equally, before definitive conclusions can be made.

#### Correlation Analysis

To supplement the descriptive information collected about the unrestricted opportunities reported as available to trainees and the number of barriers to completing those opportunities, we tested for a potential relation between the two variables. The survey questions related to those variables were multiple choice; respondents were instructed to select all that applied to them. In order to create a quantitative variable to use in a correlation analysis, we calculated the sum number of response options selected for each variable. We did not have a priori directional hypotheses about this relation.

## Independent T*-*Test Analyses

For categorical variables with mutually-exclusive response options and sufficient sample sizes, independent sample *t*-tests were used to identify potential between-group differences in the average number of different opportunities to complete unrestricted activities and in the average number of barriers for completing activities reported. Here, we report analyses for the following variables:

1. Remote supervision versus on-site supervision
2. Supervisors with trainees employed full-time versus part-time
3. Supervisors with trainees working for hourly pay versus salaried
4. Supervisors working for for-profit versus nonprofit sites
5. Supervisors who are employed by the same company as their trainees versus those who are not.

We initially planned to analyze if type of employment was associated with certain barriers or opportunities. However, the sample size of nonemployed trainees (*n* = 9) was too small to compare appropriately to employed trainees (*n* = 172).

### Qualitative Data Analysis

The final question on the survey was open-ended: “What (if any) undesirable consequences do you or your company face when providing unrestricted fieldwork opportunities for supervisees?” Authors reviewed responses and identified five primary codes to categorize within. The five, nonmutually exclusive codes were: (1) financial; (2) time to clinical permanent products; (3) time to operational permanent products; (4) performance; and (5) none listed. Operational definitions for these codes are found in Table 1. Two independent coders reviewed and coded each response. Interobserver agreement was calculated for 100% of responses. Each response included five trials for agreement (i.e., agreement was calculated for each code for each response). Agreement was calculated by dividing the number of trials the two observers agreed by the total number of trials and multiplying by 100. In cases of disagreement, the primary coder’s data were reported. Agreement was 88.89%.

**Table 1:** Qualitative codes

Code	Operational Definition
Financial	Mention of billing, loss of revenue, cost to the organization, or other financial issues
Time to clinical permanent products	Mention of increased amounts of time to completion in protocols, treatment plans, behavior plans, or any other items needed to conduct clinical services
Time to operational permanent products	Mention of increased amounts of time in coordinating schedules (trainee with client, trainee with supervisor, etc.) including schedule challenges due to stakeholder consent
Performance	Mention of burnout/stress, trainee refusal of tasks due to workload, supervisor taking on meetings outside of working hours, trainees adding to overall caseload, delay in trainee hours accrual/certification
None listed	No negative consequences listed. May include explicit responses (e.g., "N/A" or "None") or responses that did not list a negative consequence that follows providing unrestricted opportunities (e.g., "Access to clients " and "Not a part of job responsibilities and this is something that is not always understood at employment" may be considered barriers, but not consequences).

## Results

### Supervisor Demographics

Full supervisor demographic information can be found in Table 2. One hundred ninety-six individuals responded to the survey. Of those respondents, 182 reported that they did provide supervision. Their data were used in our analyses. A majority of the respondents were female (85.2%), identified as white (80.8%), and practiced in the United States (91.89%). Respondents lived in 36 different states. A majority of respondents held master’s degrees (80.8%).

**Table 2:** Respondent demographic information

Variable	N
Gender Identity	
Male	26 (14.3%)
Female	155 (85.2%)
Did not disclose	1 (0.5%)
Age range (in years)	
20–24	1 (0.5%)
25–34	56 (30.8%)
35–44	75 (41.2%)
45–54	36 (19.8%)
55–64	10 (5.5%)
65 and older	4 (2.2%)
Race/Ethnicity	
Asian	
Black or African American	4 (2.2%)
Hispanic or Latino or Spanish	9 (4.9%)
origin of any race	14 (7.7%)
White	147 (80.8%)
Two or more races	8 (4.4%)
Country	
Australia	3 (1.6%)
Canada	6 (3.3%)
Ireland	1 (0.5%)
Italy	1 (0.5%)
Spain	1 (0.5%)
United Arab Emirates	1 (0.5%)
United States	167 (91.8%)
Did not disclose	2 (1.1%)
Highest Level of Education	
Master’s degree	147 (80.8%)
Ed.S.	2 (1.1%)
Ph.D.	27 (14.8%)
Psy.D	2 (1.1%)
Ed.D	3 (1.6%)
Did not disclose	1 (0.5%)
Supervisor Qualification Category	
BCBA	155 (85.2%)
BCBA-D	25 (13.7%)
Psychologist certified by ABPP in Behavioral and Cognitive Psychology tested in ABA	1 (0.5%)

### Supervision Site Descriptive Information

Full supervision site type information can be found in Table 3. A majority of supervisors reported providing supervision on-site (75.3%) and were employed by the same company as their trainees (83.5%). A majority of respondents reported that their trainees were hourly workers (72%) and worked full-time (69.2%).

**Table 3:** Supervision site descriptive information

Variable	*N*
Supervision Provision Location	
Remote	44 (24.2%)
On location with the supervisee	137 (75.3%)
Employment Status of Supervisors and Supervisees	
Employed by same company	152 (83.5%)
Not employed by same company	30 (16.5%)
Employment Status of Supervisees	
Full time, hourly pay	87 (47.8%)
Full time, salary pay	39 (21.4%)
Part time, hourly pay	44 (24.2%)
Part time, salary pay	2 (1.1%)
My supervisees are not employed by the host site.	9 (4.9%)
Site Type	
School	60 (33.0%)
Clinic	99 (54.4%)
In-home services	92 (50.5%)
Hospital	2 (1.1%)
Community-based services	48 (26.4%)
Business consulting company	1 (0.5%)
Other	11 (6.0%)
Primary Population Served	
Children (0–21 years) with IDD^a^	148 (81.3%)
Adults (22+ years) with IDD	14 (7.7%)
Business managers and associates (i.e., OBM)	2 (1.1%)
Typically developing children (0–21 years)	5 (2.7%)
Other	13 (7.1%)
Business Model	
For-profit	105 (57.7%)
Nonprofit	57 (31.3%)
Unknown	19 (10.4%)
Did not disclose	1 (0.5%)
Payment(s) Accepted	
Private insurance	122 (67.0%)
State-funded insurance	107 (58.8%)
School district funding	53 (29.1%)
State or federal grant funding	27 (14.8%)
Private pay	54 (29.7%)
Other	11 (6.0%)

^a^ Intellectual and Developmental Disabilities

Most respondents (57.7%) reported only providing supervision at one kind of site (e.g., school, clinic, or homes only). The three most common site types reported were clinics (54.4%), in-home services (50.5%), and schools (33.3%). A majority of respondents (81.3%) reported their sites served children (0–21 years of age) with intellectual and developmental disabilities.

For-profit sites were the most-reported type of business model (57%). Thirty-one percent of respondents reported working for a nonprofit organization, and 10% did not know what kind of business model their site followed.

All but two respondents reported that their sites accept multiple sources of payment. The three most common types accepted were private insurance (67%), state-funded insurance such as Medicaid or Medicare (58.8%), and school district funding, such as that for an out-of-district placement (29.1%).

### Unrestricted Activity Opportunities Offered

Tables 4 and 5 contain complete descriptive data for the unrestricted activities respondents reported offering to trainees. All but one respondent reported offering at least one activity. Of the nine activity types provided as options, the mean number of reported different activities offered was 7.6 (range = 0–9). Each of the activities listed as options were reported as available to trainees by at least half of all respondents (mean percentage of respondents = 83.3%; range = 64.3%–89.6%). The three most commonly reported activities available were writing and reviewing behavior-analytic programs (89.6%), researching the literature relevant to a current client’s programming (89%), and observation and data collection during service delivery (89%). The least common activity reported was meeting with clients about behavior-analytic programming and services (64.3%).

**Table 4:** Unrestricted activities opportunities offered

Activity	*N*
Observation and data collection, during service delivery	162 (89.0%)
Observation and data collection, outside of service delivery	134 (73.6%)
Training staff and caregivers on behavior-analytic programs or content	155 (85.2%)
Conducting assessments related to the need for behavioral interventions	159 (87.4%)
Meeting with clients about behavior-analytic programming and services	117 (64.3%)
Conducting behavior-analytic assessments (e.g., functional analyses, stimulus preference assessments)	161 (88.5%)
Researching the literature relevant to a current client’s programming	162 (89.0%)
Writing and reviewing behavior-analytic programs	163 (89.6%)

**Table 5:** Sum number of different unrestricted activities offered

Number of activities offered	*N*
0	1 (0.5%)
1	3 (1.6%)
2	2 (1.1%)
3	4 (2.2%)
4	7 (3.8%)
5	8 (4.4%)
6	12 (6.6%)
7	25 (13.7%)
8	36 (19.8%)
9	84 (46.2%)

### Barriers to Completion

**Fig. 1:**
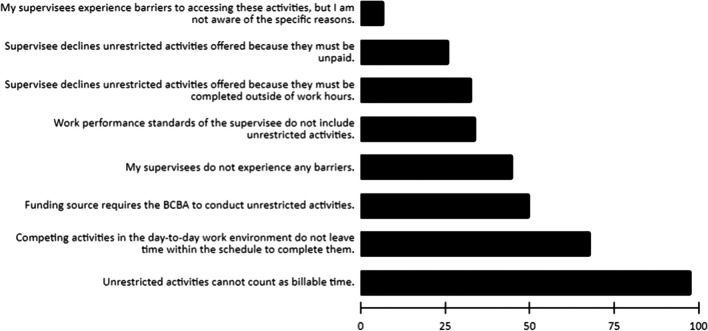
Frequency of barriers reported. *Note.* Visual depiction of the frequency of barriers reported, from least frequent (top) to most frequent (bottom)

Tables 6 and 7 contain complete descriptive data for the barriers to completing unrestricted activities respondents reported. Approximately 77% of all respondents reported at least one barrier present to completing unrestricted activities. Almost a quarter of all respondents (23%) reported there were not any barriers. The average number of barriers reported was approximately two (mean = 1.9, range = 0–7). The three most common barriers reported were “unrestricted activities cannot count as billable time” (53.8%); “work performance standards of supervisee do include unrestricted activities, but competing activities in the day-to-day work environment do not leave time within the schedule to complete them” (37.4%); and “funding source requires the BCBA to conduct unrestricted activities (27.5%). Figure 1 visually depicts how often each barrier was reported, presented from least frequent to most frequent.

**Table 6:** Barriers to completing unrestricted activities offered

Activity	N
Unrestricted activities cannot count as billable time.	98 (53.8%)
Funding source requires the BCBA to conduct unrestricted activities.	50 (27.5%)
Work performance standards of the supervisee do not include unrestricted activities.	34 (18.7%)
Work performance standards of supervisee do include unrestricted activities, but competing activities in the day-to-day work environment do not leave time within the schedule to complete them.	68 (37.4%)
Supervisee declines unrestricted activities offered because they must be unpaid.	36 (14.3%)
Supervisee declines unrestricted activities offered because they must be completed outside of work hours.	33 (18.1%)
My supervisees experience barriers to accessing these activities, but I am not aware of the specific reasons.	7 (3.8%)
Other	19 (10.4%)

**Table 7:** Sum number of different barriers reported

Number of barriers	*N*
0	42 (23.1%)
1	43 (23.6%)
2	38 (20.9%)
3	33 (18.1%)
4	14 (7.7%)
5	9 (4.9%)
6	2 (1.1%)
7	1 (0.5%)

Because there was not a way for respondents to rate each barrier by impact (i.e., they all were treated as equally important), it is possible that some respondents only selected one barrier, but the barrier is so important it overshadows other present barriers. There were 43 respondents who only reported one barrier to completing unrestricted activities. Table 8 reflects their responses. The two most common barriers from those who only selected one barrier were the same as those in the total sample: “unrestricted activities cannot count as billable time” (48.8%), followed by “work performance standards of supervisee do include unrestricted activities, but competing activities in the day-to-day work environment do not leave time within the schedule to complete them” (27.9%).

**Table 8:** Barriers selected as the only existing barrier

Activity	N
Unrestricted activities cannot count as billable time.	21 (48.8%)
Funding source requires the BCBA to conduct unrestricted activities.	4(9.3%)
Work performance standards of the supervisee do not include unrestricted activities.	1 (2.3%)
Work performance standards of supervisee do include unrestricted activities, but competing activities in the day-to-day work environment do not leave time within the schedule to complete them.	12 (27.9%)
Supervisee declines unrestricted activities offered because they must be unpaid.	1 (2.3%)
Supervisee declines unrestricted activities offered because they must be completed outside of work hours.	0 (0.0%)
My supervisees experience barriers to accessing these activities, but I am not aware of the specific reasons.	2 (4.7%)
Other	2 (4.7%)

### Post-Hoc Statistical Analyses

#### Correlation Analyses

In the total sample (*n* = 182), there was a significant, albeit small, negative correlation between the number of opportunities to complete unrestricted activities and the number of barriers for completing activities reported (*r* = -0.20, *p* < 0.05). Supervisors who reported *more* opportunities for unrestricted activities reported *fewer* barriers to completing them.

We decided to also test for a relation between the number of barriers reported and activities offered for respondents who reported there was *at least one barrier* to trainees completing unrestricted activities (*n* = 151). For this subsample, there was a significant and moderate *positive* relation identified (*r* = 0.31, *p* < 0.001). This is in contrast to the total sample. Although overall there was a negative relation between number of opportunities offered and barriers to completion, for respondents who reported there being at least one barrier, respondents who offered *more opportunities* for trainees to complete unrestricted activities also tended to report *more barriers* to completing those activities.

#### Independent T-Test Analyses

**Are there Between-Group Differences on Average Number of Different Opportunities to Complete Unrestricted Activities on any of the Tested Variables?** There were no significant differences on average number of different opportunities to complete unrestricted activities for (1) remote supervision versus on-site supervision; (2) supervisors with trainees employed full-time versus part-time; and (3) supervisors employed by the same company as their trainees versus those who are not.

There were significant differences identified in the independent groups *t*-tests for the remaining two comparisons. Supervisors working at nonprofit sites reported significantly more opportunities to complete unrestricted activities than those at for-profit sites (*t*(118.62) = -2.07, *p* < 0.05). Supervisors for salaried trainees reported significantly more opportunities to complete unrestricted activities than supervisors for hourly employed trainees (*t*(95.95) = -2.46, *p* < 0.05).

**Are there Between-Group Differences on Average Number of Different Barriers for Completing Unrestricted Activities on any of the Tested Variables?** The results for the average number of different barriers for completing unrestricted activities showed the same patterns as those for the average number of opportunities offered. There were no significant differences for (1) remote supervision versus on-site supervision; (2) supervisors with trainees employed full-time versus part-time; and (3) supervisors employed by the same company as their trainees versus those who are not.

There were significant differences identified in the independent groups *t*-tests for the remaining two comparisons. Supervisors working at for-profit sites reported significantly more barriers to complete unrestricted activities than those at nonprofit sites (*t*(160) = 3.8, *p* < 0.001). Supervisors for hourly trainees reported significantly more barriers to complete unrestricted activities than supervisors for salaried trainees (*t*(93.6) = 6.7,* p* < 0.001).

### Qualitative Analyses

Qualitative analyses were conducted for the undesirable consequences respondents reported their companies face when providing unrestricted fieldwork opportunities for trainees. Results are found in Table 9. Eighty-one respondents completed the question. The most commonly reported consequence type was financial (44.4%). This code included responses mentioning billing, loss of revenue, cost to the organization, or other financial issues. The second most commonly reported consequence type was performance-related (19.8%). This code included responses mentioning burnout/stress, trainee refusal of tasks due to workload, supervisor taking on meetings outside of working hours, trainees adding to overall caseload, delay in trainee hours accrual/certification.

**Table 9:** Coded categories of negative consequences

Code	Percentage of Responses
Financial	44.4%
Time to clinical permanent products	5%
Time to operational permanent products	9.9%
Performance	19.8%
None listed	35.8%

## Discussion

The purpose of the current study was twofold: to collect information on how qualified BCBA supervisors are currently providing unrestricted learning opportunities for their trainees and to identify barriers they encounter when offering these experiences. An analysis of the results of this study demonstrate that an overwhelming majority of respondents offer unrestricted learning opportunities to their trainees, with over half reporting offering every unrestricted activity suggested within the *BCBA Handbook* (BACB, 2022b). Despite the reporting of these activities being offered, over three quarters of respondents experience at least one barrier to providing unrestricted learning opportunities to trainees. Given that these are the activities trainees will engage in once certified and the BACB requires unrestricted activities comprise a minimum of 60% of a trainee’s fieldwork, it is important that they are able to access a large number and wide variety of related learning opportunities. This necessitates a further evaluation into potential contributing factors of any limitations and how to avoid such limitations. Within the current study, we identified potential factors related to fewer barriers, such as nonprofit business models and salaried trainee employees, as well as potential factors related to increased barriers, such as for-profit business models and hourly trainee employees.

Supervisors that reported working in a nonprofit business model reported being able to offer more unrestricted learning opportunities to their trainees than those in for-profit business models. In addition, supervisors in nonprofit business models reported fewer barriers in offering these learning experiences than those in for-profit business models. In a similar pattern, supervisors whose trainees were salaried employees reported offering more unrestricted learning opportunities to trainees and reported fewer barriers to offering these activities than those with hourly employees. In general, for-profit business models operate with a single goal: to turn a profit. Nonprofit business models differ from for-profit models in that they are mission-driven, focused on public benefit, and only use profits to advance the organization (U.S. Chamber of Commerce, 2021). That is, profits made within this model ideally go towards funding continued service. Business practices that support continued service cannot guarantee good quality service delivery and related training. However, these findings suggest that the current practices within nonprofit service provision may be more conducive to meeting the training standards set forth by the BACB (2022b), providing, at minimum, the opportunity for supervisors to provide good quality supervision and unrestricted learning opportunities to trainees.

Employment status and profit model of your organization are both inextricably tied to financial contingencies; for example, a company’s profit model is focused on either profit or mission, and choosing to provide a salaried position rather than an hourly one means a company is choosing to provide a predetermined amount of pay on a regular basis, rather than as services are rendered (U.S. Chamber of Commerce, 2021; U.S. Department of Labor, 2019). The current findings are important particularly when considering two of the three most common reported barriers by supervisors were financial; in particular, restrictions on billing practices from funding sources. Furthermore, the most commonly coded undesirable consequence reported by supervisors was also financial, with responses mentioning billing, loss of revenue, cost to the organization, or other financial issues. Financial factors being reported by supervisors as a barrier to providing trainees unrestricted learning opportunities may be an example of the effects of ABA service delivery occurring largely within the capitalist health-care system in the United States. Garner et al. (2022) explores the contingencies ABA service delivery is subject to within this system. Their discussion focuses on “. . . contingencies that promote freedom, democracy, and cooperation as well as the contingencies that promote greater exploitation, less freedom, and more coercion” (p. 174). Within this, special focus is on for-profit business models, particularly those within private equity. Since 2004, the number of ABA service delivery companies operating within a private equity-backed, for-profit business model has grown considerably (for a full review, see Olson, 2022). According to Garner et al.’s findings, the effects of a private equity business model on applied behavior-analytic service delivery are as of yet unstudied. However, findings in other health-care disciplines such as nursing suggest the potential for negative effects, chiefly among them, poor clinical outcomes (e.g., Gupta et al., 2021). Fortunately, Garner et al. (2022) also propose actionable solutions to such issues. Some of these include support from our professional organizations in the form of tools which calculate an appropriate practitioner workload.

Although this recommendation was intended to help prevent poor clinical practices, it could also affect quality of supervision and training. If supervisors have an appropriate workload that is accounted for within the financial planning of the organization, trainees may be more likely to be exposed to better clinical practices and may be provided more unrestricted learning opportunities as a result. Future researchers should evaluate both the effect private equity business models have on provision of clinical services and quality of training provided to future practitioners.

The fields of psychology and medical education include guidelines for their supervised experiences similar to those provided by the BACB and have to operate within the same capitalist health-care system in the United States. One distinction that sets the requirements of these fields apart from the requirements of the BACB is that they include supervised training both during and following the completion of the trainee’s education. In the case of medical doctors, completing the majority of their supervised training upon the completion of their degree allows for the provision of a license to practice. Likewise, in some states, postdoctoral psychologists acquire a provisional license. Because these licenses are regulated by the states in which the trainees are completing their supervision experiences, the issues with third-party payers may be reduced compared to situations in which the trainee does not possess such a license, providing these practitioners with more opportunities for supervised experience. Future researchers should evaluate the extent to which a provisional credential (e.g., state license or national certification) and allowable billing could alleviate the financial barriers incurred while trainees acquire unrestricted learning opportunities.

Within the whole sample, supervisors who reported more opportunities for unrestricted activities reported fewer barriers to completing them. However, when we removed supervisors that reported no barriers to offering these opportunities, we see that the subsample of supervisors that offer more activities actually tend to experience more barriers. When more barriers are experienced, the likelihood for tasks to be left unfinished may increase; the trainee may not complete the task at all, or, if they do complete the task, the supervisor may not be able to provide them with necessary feedback required for repertoire shaping, etc. This could contribute to problems in the quality of a trainee’s experience; when learning opportunities are limited in scope, the ability to acquire the many different skills required by a BCBA also becomes limited. If contingencies select for supervisors to limit the number and quality of training experiences provided to trainees, the supervisor’s ability to adhere to the ECBA (BACB, 2022c) comes into question. In particular, section 4.06, “Providing Supervision and Training” of the ECBA covers a behavior analyst’s responsibility to provide positive reinforcement-focused, individualized, and evidence-based training within supervision. Provision of unrestricted learning opportunities is inherent in this section.

Previous literature on supervision practices offers potential solutions to this ethical challenge. For example, Hartley et al. (2016; 2023) proposes an apprenticeship model to meet the BACB’s supervision standards. Within this model, which was in place at the authors’ organization at the time of publication, trainees who have completed all of their restricted hours act as apprentices and are expected to shadow their BCBA supervisor and become fluent in the job responsibilities required of a BCBA, such as clinical case oversight, performance management of staff, client treatment planning, and clinical operations tasks. Trainees working as apprentices receive monthly performance evaluations and three forms of weekly or biweekly supervision: one focused on client interactions, one focused on review of the trainee’s client-related permanent products (e.g., designed data sheets), and one client team meeting focused on the trainee’s supervisory skills. These opportunities for skill-building align with the standards set forth by the ECBA, 5^th^ edition *Task list*, and the 6^th^ edition *TCO* (BACB, 2022c, 2017b, 2022a).

One may assume such a labor-intensive model is financially burdensome. Indeed, many of the tasks of the apprentice (and the BCBA) are not billable to third-party payers, based on the available CPT Codes (AMA, 2018), and therefore, cannot be directly tied to financial returns. However, Hartley et al. (2016) reported this model to be financially beneficial to their organization. In particular, an apprentice allows for increases to the caseload capacity for their BCBA supervisor. With an increased caseload, Hartley et al. reported “. . . a financial benefit to the organization of $475,000 to $500,000 annually” (p. 335). Given the specific learning opportunities for trainees and potential financial benefits to companies associated with this model, apprenticeships may provide an actionable solution to reducing barriers to offering unrestricted learning opportunities, regardless of the business model a BCBA supervisor works within. In addition to the financial and training solutions, the apprenticeship model may also help to combat recent trends in burnout in ABA clinicians (e.g., Plantiveau et al., 2018). In the current study’s qualitative analysis, the second-most reported negative consequence coded was performance-related, noting things such as supervisor burnout, stress, and taking on additional, uncompensated work. If additional support from a trainee and the trainee’s supervision are preplanned within the work performance standards of a supervising BCBA and their apprentice, there is potential for better work–life balance and less burnout.

Overall, a number of findings with future implications were identified in the current study. However, our analysis did not provide information for all factors investigated. Factors the current study investigated, but did not identify as potentially important for availability and barriers to unrestricted learning opportunities included: whether supervision was provided primarily remotely or primarily on site, whether trainees worked full time or part time, and whether the supervisor was employed by the same site as the trainee. Though additional research needs to be conducted before final conclusions can be made about these factors, promising hypotheses can be generated. For example, supervision provided remotely may be far more accessible than that which is conducted in-person. If unrestricted learning opportunities are not affected by the mode of supervision, and quality guidelines for remote supervision are followed, this could provide support for increased use of remote supervision; a more widely available alternative. Future researchers should evaluate the extent to which modality of supervision, employment status of the trainee, supervisor affiliation affect the provision of unrestricted learning opportunities using a-priori hypotheses and participants recruited to reflect the desired subgroups.

### Future Directions and Limitations

The current study highlights opportunities for future research, given some limitations. First, this is the first study to explicitly study the unrestricted learning experiences offered to trainees in behavior analysis. Therefore, the study should be replicated. Second, although the focus of this article was on the unrestricted learning opportunities of trainees in behavior analysis, data were gathered from the supervisors of the trainees. Future research should expand the participant pool to include the trainees themselves, because they likely have specific information and insight that differs from their supervisors. Finally, the current study recruited supervisor participants based on their status as a qualified BCBA supervisor (BACB, 2022b), with the majority of respondents residing in the United States. Given that the unrestricted hours requirements to become a BCBA applies to the authorized countries of the United States, Canada, Australia, and the UK (BACB, 2023), the current study’s data may not represent the other three countries. Future researchers should seek respondent participation from all authorized countries to provide a more complete illustration of the current practices and barriers in accessing unrestricted learning opportunities. Furthermore, individuals seeking certification outside of these countries may do so as an international behavior analyst (IBA; International Behavior Analysis Organization [IBAO], 2021). Although the requirements to acquire the BCBA and the IBA credentials differ (e.g., number of practice hours, graduate degree necessity), experiences of supervisors with an IBA may be helpful in creating a well-rounded discussion of the training needs of the field at-large. Future research should survey the training practices and barriers of IBA supervisors as a means of starting a more global discussion on training in behavior analysis.

This study also provides future opportunities from a methodological perspective. In particular, our quantitative analyses were designed as supplemental rather than the focus of this study, and thus, we had no a priori directional hypotheses. The results are post-hoc and could be sample specific. Thus, our analyses need to be replicated with a priori directional predictions before more conclusive statements can be made.

Next, we did not recruit samples at the onset of the project to equally represent each subgroup analysis. This resulted in some subgroups having relatively different sample sizes in our independent groups *t*-test comparisons. It is important to note that equal subgroups are not an assumption of *t*-tests, and we did correct for unequal variances in our analyses when necessary. However, these analyses need to be replicated with intentionally recruited samples to produce more definitive conclusions.

Finally, the questions related to unrestricted activities available and barriers present treated all response options as unweighted. Thus, regardless of the impact of a single barrier, or the frequency of the activity’s availability, any response was weighted as equally important or available. For example, one respondent might report two unrestricted activity types available, but those activities are available to every trainee multiple times a week. Another respondent might report there are six different activity types available, but those activities are only available to a few trainees once a month. The former respondent’s “score” for the sum number of activities offered would appear lower than the latter, even though arguably the availability of unrestricted activities is higher in quality than the second respondent. For barriers, perhaps one respondent only reports one barrier present: unrestricted hours cannot count as billable time. There may be other barriers present, but to the respondent, this barrier supersedes all other barriers. As an alternative, perhaps a respondent reports five different barriers, but there is one primary barrier that is the most important. The other four, although present, do not really affect the day-to-day availability of unrestricted activities. Although both respondents may experience similar realities, their “scores” for barriers present are different. Future studies should either clarify that respondents are to select the most important barrier, or, allow respondents to rate the impact of each barrier. This would allow a more complete analysis of the potential effect of certain barriers on availability of unrestricted activities. Although limitations to our analyses are present, the results of these analyses do serve a hypothesis-producing function. We intend for these results to spur further discussion of the availability of unrestricted activities and barriers to completing them at present.

## Conclusion

As of 2021, the demand for BCBAs had increased by 5852% since 2010 (BACB, 2022d). This increase in demand for behavior analysts inadvertently increases the demand for high quality training, including those focused on unrestricted activities. The findings of the current study suggest opportunities for high quality training exist, but so too do barriers to creating these training opportunities. We hope that our field will continue to investigate how to reduce barriers to unrestricted learning opportunities using these findings and those of other supervision-focused studies. Through that, future generations of behavior analysts will have the space to build repertoires that benefit not only the science, but society as a whole.

## Declarations

### Data Availability

The datasets generated during and/or analyzed during the current study are available from the corresponding author on reasonable request.
